# Trivial trauma and delayed rupture of a normal spleen: a case report

**DOI:** 10.1186/1752-1947-5-591

**Published:** 2011-12-21

**Authors:** Nicholas Sowers, F Kris Aubrey-Bassler

**Affiliations:** 1 Emergency Medicine, Dalhousie University, Halifax, NS, Canada; 2 Primary Healthcare Research Unit, Memorial University of Newfoundland, St. John's, NL, Canada

**Keywords:** spleen, splenic rupture, wounds and injuries, delayed diagnosis, minor trauma, delayed splenic rupture

## Abstract

**Introduction:**

Although a majority of splenic ruptures present acutely with a known mechanism of injury, a minority of patients present days to weeks following trauma with a delayed rupture. Also uncommon is the atraumatic rupture, the vast majority of which occur in patients with underlying splenic pathology. A handful of cases of apparently spontaneous rupture of a normal spleen are reported; however, there is debate about whether these actually represent delayed ruptures following a history of trauma that is not elicited. Although a few cases of delayed rupture of the spleen following trivial trauma have been reported, the majority of these present evidence of an underlying disease process. We found only two such cases that documented a normal spleen and three cases where underlying splenic pathology was not reported. We review the literature and discuss the phenomenon of delayed rupture of the normal spleen following trivial trauma.

**Case presentation:**

A 27-year-old Caucasian man with no underlying splenic pathology presented with splenic rupture one week after playfully wrestling with his partner. The patient did not present at the time of the injury and only recalled it upon repeated questioning after computed tomography diagnosis.

**Conclusions:**

This case lends support to the theory that the normal spleen can rupture some time after trivial trauma, which seems like a more plausible explanation than rupture without cause. However, given the dearth of similar reports in the literature, the possibility remains that the association we have observed is not causational.

## Introduction

In patients sustaining blunt abdominal trauma, the spleen is the most commonly damaged viscus [1]. Although a majority of patients with this injury present acutely, up to 15% present with a delayed rupture days to weeks following a substantial abdominal injury. The mortality for patients presenting with acute splenic rupture is approximately 1% whereas the mortality associated with delayed rupture approaches 15% [2]. While atraumatic rupture of the spleen is not unheard of, cases reported in the literature of such a rupture are rare and usually occur in a diseased spleen [3].

There is debate in the literature as to whether apparently atraumatic ruptures of the spleen are truly spontaneous or actually represent a delayed rupture following a history of trauma that is not elicited at the time of presentation (reviewed in [3]). In 1958, Orloff and Peskin proposed four criteria to define what they refer to as 'spontaneous' rupture of a spleen [4], which emphasizes that the spleen must appear grossly and histologically normal. In the same paper, they cite 71 reports documenting ruptures of the spleen labelled as spontaneous, only 20 of which fulfilled all of their criteria. Thus, usage of the term spontaneous was inconsistent and continues to be so in the more recent literature, with many authors labeling the rupture of diseased spleens as spontaneous.

We present the case of a 27-year-old Caucasian man without risk factors for rupture and an apparently normal spleen who presented with a delayed rupture and significant hemoperitoneum one week after sustaining a left sided abdominal injury. The injury was so minor that the patient did not present for assessment at the time and only recalled the incident following radiological diagnosis and subsequent questioning. We found only two cases of delayed rupture of normal spleen following trivial trauma reported in the literature in the last 60 years. In one case, the spleen was enlarged [5] and in the other, splenic size and weight were not reported [6]. Three other reports of delayed rupture following such trauma published in the same time period did not include information on evidence of splenic disease [7-9]. Given that the spleen was enlarged in the first case, not reported in the second case and that disease information was not reported in the other three, the possibility remains that all of these represent ruptures of diseased spleens, and that our case is the first report of delayed rupture of a normal spleen following trivial trauma. Given the dearth of publication in this area, the possibility remains that the associations observed in these reports are coincidental rather than causal. However, we feel that documenting these cases may lead to improved recognition of similar cases in the future and possibly to an enhanced understanding of the pathophysiology of splenic rupture.

## Case Presentation

A 27-year-old Caucasian man presented to the emergency department (ED) following the acute onset of severe, sharp chest and abdominal pain radiating to his shoulder blades and testicles. These symptoms began acutely five hours earlier, waking him from sleep and were associated with nausea and generalized weakness. He was initially able to return to sleep although he clearly stated that the pain was exacerbated in the supine position. On presentation to the ED, his initial vital signs included a blood pressure of 80/60 mm Hg and a heart rate of 60 beats/minute described as 'thready.' His respiratory rate was 26 per minute and he was afebrile. His blood pressure while supine prior to fluid resuscitation was 98/60 mm Hg. The patient had a single episode of emesis in the ED. The initial abdominal examination demonstrated both guarding and rebound tenderness with normal bowel sounds but the remainder of the physical examination, including the testicles, was unremarkable. Fluid resuscitation was initiated and blood pressure stabilised at 110/60.

His past medical history and review of systems were unremarkable including no peptic ulcer disease and no travel history. Family history did not include any bleeding diathesis, connective tissue or rheumatologic condition. His only medication was ranitidine recently taken for heartburn as needed.

Initial blood work demonstrated a hemoglobin level of 115 g/L without any obvious history of bleeding and a white blood cell count of 13.8 g/L. Liver enzymes, electrolytes, blood glucose, blood urea nitrogen, creatinine, and amylase were essentially normal, and an electrocardiogram was unremarkable. Both the ED physician and consultant radiologist reported erect and supine views of the abdomen and postero-anterior and lateral views of the chest were normal. In particular, no rib fractures were evident on the chest X-ray, and the left hemi-diaphragm appeared normal. The point of care ultrasound machine was out-of-service at the time this patient presented.

**Figure 1:**
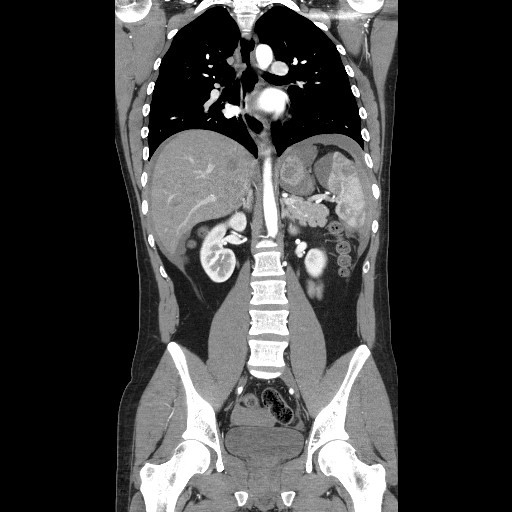
**Coronal computed tomography image demonstrating macerated spleen, perisplenic hematoma and hemoperitoneum**.

Intense pain persisted after a total of 20 mg of morphine, 50 mg of dimenhydrinate and 20 mg of hyoscine. He was sent for a contrast enhanced computed tomography (CT) scan of the chest, abdomen and pelvis which demonstrated a macerated spleen with rupture resulting in a significant hemoperitoneum (Figure 1). Only after this revelation and with repeated specific questioning did the patient recall an apparently trivial injury to his left side about one week prior to presentation while playfully wrestling with his partner. He did not present for assessment of the injury at that time.

Splenectomy and post-operative recovery were uneventful and he was discharged home one week after presentation. The pathology report of our patient's spleen later documented an organ weighing 235 grams and measuring 14.0 × 9.5 × 5.5 cm. This weight is within the reported range of normal [10]. Approximately 30% of the splenic parenchyma contained dilated hemorrhage filled vascular areas but the uninvolved parenchyma appeared grossly and histologically normal.

## Discussion

Delayed rupture of the spleen following significant trauma is relatively rare but is well documented in case reports, series and textbooks. A review of reported cases demonstrates that the vast majority of patients with normal spleens ultimately diagnosed with delayed rupture sustained an injury of such significant magnitude that it required initial assessment in the emergency department. Previously described mechanisms have included automobile, motorcycle, or all terrain vehicle crashes, violent assaults or falls from a height (reviewed in [11]). Atraumatic ruptures of the spleen are also well described in spleens diseased by hematologic malignancy, parasitosis, infiltrates and in patients with impaired coagulation (reviewed in [3]). We present one of only a very few reported cases of delayed rupture of the normal spleen temporally associated with a trivial traumatic insult.

The hallmark presentation of a patient with splenic rupture includes left upper quadrant pain, Kehr's sign (shoulder pain secondary to diaphragmatic irritation by the hemoperitoneum) [1,2], as well as signs of generalized peritonitis such as guarding and rebound tenderness. Depending upon the severity and duration from onset to presentation, patients may also present with signs of hemodynamic compromise. Given this relatively nonspecific presentation there is often a wide differential diagnosis including much more common conditions such as appendicitis [1], acute coronary syndrome, gastric ulcer, renal colic and abdominal aortic aneurysm. Considering the difficulty in making the diagnosis of delayed splenic rupture, keen clinical suspicion on the part of the emergency room physician needs to be supported with the appropriate use of various imaging modalities such as point- of-care ultrasound and computed tomography (CT). The accuracy of computed tomography (CT) for diagnosis of splenic injury is approximately 97% [2]. Thus, it is tempting to consider the use of abdominal computed tomography (CT) in a patient with unexplained abdominal pain even if they are hemodynamically stable, give no history of injury, and have an unremarkable ultrasound. However, we are cognisant of the recent dramatic increase in computed tomography (CT) utilization and the potential hazards associated with it and thus would not make such a blanket recommendation.

It is interesting to note that despite significant intra-abdominal blood loss and hypotension, our patient's heart rate remained stable at < 90 bpm throughout his stay in our emergency department. Similarly, cases reported in the literature also describe relative bradycardia [2,12]. This phenomenon has previously been described and is likely explained by diaphragmatic and vagal nerve irritation by intra-abdominal blood [13]. In addition, both our patient and another reported in the literature [12] complained of gastro-esophageal reflux (GER) prior to presentation. We are unaware of a relationship between GER and splenic rupture; however, it may be that the symptoms diagnosed as GER in these cases may actually have been attributable to an evolving splenic injury.

## Conclusion

It seems more plausible that a non-diseased spleen should rupture as the result of some traumatic insult, even if temporally removed from the time of presentation, than spontaneously. However, only a handful of cases potentially compatible with this explanation are reported in the literature, while a larger number of cases reported as spontaneous (and ostensibly meeting Orloff and Peskin's criteria [4]) have been reported. The possibility remains that the association we have observed is not causal. However, given the non-specific symptoms associated with splenic rupture, the relatively high mortality rate and the effectiveness of early therapy it would be prudent to ask patients presenting with non-specific abdominal pain in detail about major and minor trauma in the preceding weeks. Eliciting such a history could lead to more timely diagnosis and therefore to a lower mortality rate.

## Consent

Written informed consent was obtained from the patient for publication of this case report and any accompanying images. A copy of the written consent is available for review by the Editor-in-Chief of this journal.

## Competing interests

The authors declare that they have no competing interests.

## Authors' contributions

NS and KA were involved in literature searching, interpretation of the case, manuscript revision and final approval. NS wrote the first draft of the paper. Both authors read and approved the final manuscript.
